# Environmental Hazards and Chemoresistance in OTSCC: Molecular Docking and Prediction of Paclitaxel and Imatinib as BCL2 and EGFR Inhibitors

**DOI:** 10.3390/biology14091174

**Published:** 2025-09-02

**Authors:** Nishant Kumar Singh, Prankur Awasthi, Agrika Gupta, Nidhi Anand, Balendu Shekher Giri, Saba Hasan

**Affiliations:** 1 Amity Institute of Biotechnology, Amity University Uttar Pradesh, Lucknow 226028, India; 2 Department of Pathology, Dr. Ram Manohar Lohia Institute of Medical Science, Vibhuti Khand, Gomtinagar, Lucknow 226010, India; 3 Sustainability Cluster, University of Petroleum and Energy Studies (UPES), Dehradun 248007, India

**Keywords:** OTSCC, EGFR, BCL-2, environmental pollutants, chemotherapy, irinotecan

## Abstract

Oral tongue squamous cell carcinoma (OTSCC) is a common type of oral cancer influenced by genetic, epigenetic, and environmental factors like exposure to environmental toxins. These environmental toxins can decrease the effectiveness of established chemotherapy drugs, such as Irinotecan, used in OTSCC treatment. Bioinformatics, drug discovery, and machine learning techniques were employed to investigate the impact of Irinotecan on OTSCC patients by identifying targets and signaling pathways, including those that positively influence protein phosphorylation, protein tyrosine kinase activity, the PI3K-Akt (Phosphatidylinositol 3-kinase- Protein Kinase B) signaling system, cancer pathways, focal adhesion, and the HIF-1 (Hypoxia-Inducible Factor 1) signaling pathway. Later, the protein–protein interactions (PPIs) network, along with twelve cytoHubba approaches to finding the most interacting molecule, was employed to find the important proteins BCL2 and EGFR. Drugs related to BCL2 and EGFR were extracted from the DGIdb database for further molecular docking. Molecular docking revealed that Docetaxel, Paclitaxel, Imatinib, Ponatinib, Ibrutinib, Sorafenib, and Etoposide showed more binding affinity than Irinotecan (i.e., −9.8, −9.6). Of these, Paclitaxel (−10.3, −11.4) and Imatinib (−9.9, −10.4) are common in targeting BCL2 and EGFR. Using these identified candidate genes and pathways, we may be able to uncover new therapeutic targets for the treatment of OTSCC. Furthermore, molecular dynamics (MD) simulations were performed for selected ligand–receptor complexes, revealing stable binding interactions and favorable energetic profiles that supported the docking results and strengthened the reliability of the proposed drug repurposing strategy.

## Simple Summary

Oral tongue squamous cell carcinoma (OTSCC) is a type of mouth cancer that can be caused by both inherited and environmental factors, such as exposure to harmful substances. Some drugs, such as the chemotherapy medication Irinotecan, can reduce the effectiveness of common treatments. This study utilized computational methods to investigate the genes and proteins associated with OTSCC to better understand the underlying molecular mechanisms and therapeutic responses. The analysis identified two critical proteins, BCL2 (B-cell lymphoma 2) and EGFR (Epidermal Growth Factor Receptor), as being significantly involved in the progression and spread of OTSCC. To identify more effective treatment options, alternative drugs were screened for their ability to bind to these target proteins using molecular docking techniques. Seven compounds demonstrated a stronger binding affinity than Irinotecan, with Paclitaxel and Imatinib showing the ability to target both BCL2 and EGFR effectively. To strengthen these findings, molecular dynamics simulations were conducted, which confirmed the stability and favorable interaction of these drug–protein complexes over time, reinforcing their potential as repurposed therapeutic agents for OTSCC. This study suggests that these drugs may be better options for treating OTSCC and offers hope for developing new and more effective treatments in the future by focusing on these target proteins and pathways.

## 1. Introduction

Cancer is the fastest-growing cause of death worldwide, having caused a predicted 10 million deaths in 2020 (1 in every 6 deaths) [1]. According to previous research, some reasons for poor air quality include emissions from power plants, vehicles, and residential burning [2,3]. Additionally, a wide range of epigenetic modifications, including DNA methylation, 5-hydroxymethylation, post-translational modification of histones, and altered gene expression, have been linked to outdoor air pollution [4]. As a result, several types of body malignancies, including cancers, have been linked to outdoor air pollution.

Oral cancer ranks as the 11th most prevalent cancer globally and is the leading cause of head and neck cancer (HNC), having a significant death rate due to late detection and early metastasis [5]. Heavy metals in the environment, such as arsenic (As), nickel (Ni), and chromium (Cr), are some examples of cancer risk factors [6,7]. Oral tongue squamous cell carcinoma (OTSCC) is one of the particularly strongly prevalent forms of oral cancer, with a significant tendency for invasion and metastasis [8,9]. According to Siegel et al. (2018), OTSCC is the sixth-fastest-growing cancer in the oral cavity and develops in the anterior two-thirds of the tongue [10]. With an increased rate of recurrence and an unremarkable improvement in 5-year survival, it is an aggressive HNC [11]. In the most severe and recurring cases, chemotherapy (Irinotecan, 5-fluorouracil, taxol, and carboplatin or cisplatin) usually results in negative impacts and death shortly after a year of recurrence/relapse, since the patient no longer responds to therapy [12,13].

**Figure 1:**
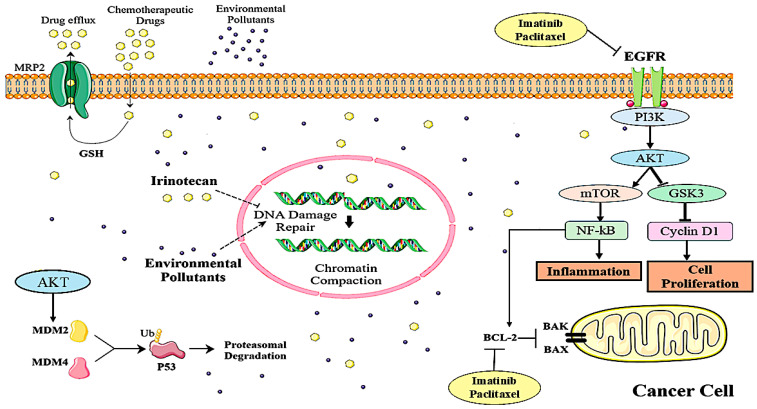
Environmental pollutants promote chemoresistance by activating survival pathways (PI3K/AKT/mTOR and NF-κB), enhancing drug efflux, DNA repair, and p53 degradation.

The efficacy of several common chemotherapy medications may be decreased by a number of environmental contaminants, which could result in cancer patients developing chemoresistance [14]. However, the accurate diagnosis of and therapy to treat OTSCC depend on an understanding of gene expression. According to Yuan et al., 2020, a number of dysregulated genes and disease processes may have an impact on the development of OTSCC [15]. By understanding these pathways and dysregulated genes, we can develop therapeutic strategies and a better understanding of the disease [15]. Some genes, like EGFR, TCRP1 (Tongue Cancer Resistance-Associated Protein 1), ZEB1 (Zinc Finger E-box Binding Homeobox 1), BCL-2, MACC1 (Metastasis Associated in Colon Cancer 1), NRLP3 (NOD-like Receptor Family Pyrin Domain Containing 3), and PAK1 (p21 (RAC1) Activated Kinase 1), are associated with the mechanism of chemoresistance development in OTSCC [16]. This correlates with a study by Lagunas-Rangel et al., 2022, in which they studied the molecular mechanism behind the development of chemoresistance through environmental pollutants [14]. Pollutants such as persistent organic pollutants, benzo[a]pyrene, Aluminum Chloride, bisphenol A, and Airborne Particulate Matter activate signaling pathways like NF-κB (Nuclear Factor kappa-light-chain-enhancer of activated B cells), ERK/MAPK (Extracellular Signal-Regulated Kinase/Mitogen-Activated Protein Kinase), and PI3K/AKT. These pathways are favorable to survival, proliferation, cell cycle progression, the suppression of p53 signaling, inflammation, migration, and invasive DNA damage reduction via the compaction of chromatin, enhanced repair, and drug efflux and antioxidant enzyme action, which leads to a decrease in the action of chemotherapeutic drugs and leads to chemoresistance [14] (Figure 1). Henceforth, targeting less effective chemotherapeutic drugs would be the better strategy to understand the related mechanisms and hence improve their efficacy.

The topoisomerase I inhibitor Irinotecan (IRN), which has the potential to treat solid tumours such as metastatic lung and colorectal cancer, was chosen as a decreased chemotherapeutic drug [17,18]. IRN has shown some therapeutic effect in patients with metastatic or recurrent HNSCC (R/M HNSCC) [19,20]. Yet, some studies showed that patients with OTSCC still relapse on Irinotecan [12,13]. Therefore, it is necessary to combine this drug with another to improve the efficiency of treatment. Targeting the genes associated with IRN in OTSCC could be a better approach to improve therapy for OTSCC patients.

In the current study, an integrative bioinformatics analysis was conducted, and strong DEGs linked to OTSCC carcinogenesis were searched in the GEO database. A network of protein–protein interactions (PPIs) was established to uncover major hub proteins. The KEGG and the GO were used for enrichment analysis to determine the key targets. Finally, we identified other approved drugs to target hub proteins through molecular docking to see the effectiveness of other drugs with respect to Irinotecan. To further validate the docking outcomes and assess the dynamic stability of ligand–protein complexes, molecular dynamics (MD) simulations were also performed, which revealed favorable interaction stability and energy profiles. In addition, by providing accurate molecular biomarkers for screening, diagnosis, and therapy planning, our research may aid in identifying the drugs used to treat OTSCC. This study would be helpful in sustainable healthcare management.

## 2. Materials and Methods

### 2.1. Microarray Data

The following parameters were used to search the GEO database: Study type: “Expression profiling by array”; search term: “OTSCC”. The analysis used two datasets, GSE78060 [21] and GSE138206 [22], both of which were based on the GPL570 platform. The dataset GSE78060 had 26 samples of tongue cancer and 4 normal samples. Subsequently, dataset GSE138206 contained 5 samples of tongue cancer and 5 normal samples (Table 1).

### 2.2. Identification of DEGs

#### 2.2.1. OTSCC-Related DEGs

An interactive web tool called GEO2R from the Limma v 3.54.0 package of R software v 4.2.2 was used to analyze the original datasets obtained from GEO. The cutoff parameters for DEG analysis were |log FC| in the range of +1 to −1, adjusted *p*-value < 0.05. Messenger RNAs (DEGs) were expressed differently in OTSCC, and normal tissues were looked for using GEO2R. The two datasets screened for different expression genes were then combined through the SRplot tool (https://www.bioinformatics.com.cn/en) accessed on 4 March 2025 to produce a volcano plot of DEGs.

#### 2.2.2. Irinotecan-Related DEGs

Various types of databases, including Drug Bank (www.drugbank.ca), the Swiss Target Prediction database (https://www.swisstargetprediction.ch/), and STITCH (http://stitch.embl.de/), are utilized to detect potential Irinotecan targets. Details on drugs regarding targets, interactions, and their effects are available in the sophisticated and freely accessible online database known as Drug Bank (www.drugbank.ca). To generate a complete profile of interactions, STITCH (http://stitch.embl.de/) combines data from binding experiments, crystallized structures, metabolic pathways, and interactions of drug targets. Swiss Target Prediction (http://www.swisstargetprediction.ch) may be used to identify bioactive component targets by contrasting 2D and 3D homology qualities with confirmed ligands.

### 2.3. Filtration of Common DEGs Between OTSCC and Irinotecan

After evaluating all DEGs, Venn plots were produced between these datasets using Venny 2.1 (https://bioinfogp.cnb.csic.es/tools/venny/), accessed on 4 March 2025, to determine the common genes connected to Irinotecan. Frequently, Venn diagrams are employed in the scientific community to illustrate the differences between gene lists produced by various differential studies. Volcano plots have been developed to identify common DEGs considering |log FC| in the range of +1 to −1 and an adjusted *p*-value < 0.05.

### 2.4. An Examination of Functional and Pathway Enrichment

The GO Functional Enrichment study of DEGs allows for the computational evaluation of biological systems within the molecular, cellular, and organismal levels in a broad variety of species. They are referred to as the biological process (BP), molecular function (MF), and cellular component (CC), respectively. The KEGG is a high-level biological system that utilizes an operational database that depends on molecular-level data from genome sequencing and other high-throughput experimental techniques. DAVID 6.8 (https://david.ncifcrf.gov) offers an extensive set of functional annotation tools for investigating gene group functional enrichment. DAVID assesses GO performance and KEGG pathway enrichment to determine the biological importance of DEGs in oral cancer, using a *p* < 0.05 enrichment threshold as a cut-off criterion. The bubble plots were created using SRplot after the data from the GO enrichment and KEGG analyses were analyzed.

### 2.5. PPI Networks to Select Potential Key Genes

Protein–protein interaction (PPI) analysis of networks is widely used to examine the underlying causes of diseases and identify novel therapeutic targets. The STRING v12.0′s database (https://string-db.org) connects over 5000 species both directly (physically) and indirectly (functionally) through PPI. Cytoscape v3.9.1 was used to examine the results of STRING v12.0′s PPI analysis of DEGs. In our investigation, different algorithms, like DMNC, Stress, MCC, Degree, EPC, Closeness, Clustering Coefficient, MNC, Betweenness, Bottleneck, Eccentricity, and Radiality, were used to identify hub genes.

### 2.6. Identification for Drugs Related to Key Genes and Molecular Docking Analysis

To predict drugs that will target key genes, the Drug-Gene Interaction Database (DGIdb) (http://www.dgidb.org) was employed. The DGIdb functions as a hub for data collecting on drug–gene interactions and drugability from a variety of sources [23]. Drugs that were approved by the FDA were chosen for the docking analysis. In the network of protein–protein interactions, the most crucial proteins may be used for further molecular docking research [24]. Docking is a drug design technique that estimates the preferred arrangement of a particular molecule to another as they combine to form an equilibrium complex. The three-dimensional structure of the drug was established using the database PubChem and control inhibitors of the target proteins [25]. The RCSB Protein Data Bank supplied the protein receptors BCl2 (PDB ID: 6o0k) and EGFR (PDB ID: 1xkk). All molecular docking studies were carried out using Auto Dock Vina software v 1.5.7. An open-source molecular docking Programme called Auto Dock Vina can dock many ligands simultaneously and includes a score system [26]. By removing hetero atoms, cofactors, and water molecules from the hub proteins and ligands in Molecular Graphics Laboratory (MGL 1.5.6), Auto Dock Vina tools were used to develop the 3D format [27]. Using Discovery Studio, the outcomes of docking with the lowest possible binding energy were then visualized.

### 2.7. Molecular Dynamics Analysis

To learn more about the behavior of the docked ligand–protein complexes, molecular dynamics simulations were employed. The generic AMBER force field (GAFF2) and the OpenMM engine on the Google Colaboratory platform were used to run the simulations [28] based on the protocol outlined by Arantes et al. [29], with a few modifications, as outlined below.

PDB files were put into the Google Colab notebook in order to create the ligand and protein topologies. The TIP3P water model was then used to solvate the systems after periodic boundary boxes were positioned 12 Å from the atoms. Sodium chloride (NaCl) ions were introduced at a concentration of 0.15 M to neutralize the system’s overall charge. The solvated systems underwent energy minimization for 20,000 steps, followed by a five-nanosecond equilibration using a two-femtosecond time step under NPT ensemble conditions, maintaining a constant number of atoms, 1 bar of pressure, and a temperature of 298 K. At 10-picosecond intervals, snapshots were taken during the equilibration stage. After equilibration, 100 ns of molecular dynamics simulations (MDs) were run using the NPT ensemble settings, keeping the temperature at 298 K and the pressure at 1 bar constant. Every 100 picoseconds, trajectory files (.dcd files) were created. Following molecular dynamics simulations, trajectory files were analyzed using a Python-based pipeline with MDAnalysis and py3Dmol to extract solvent-free atomic coordinates and generate an interactive 3D animation of conformational changes over time. Various properties, including MM/GBSA, root mean square fluctuation (RMSF), root mean square deviation (RMSD), and radius of gyration (RoG), were calculated from the trajectories.

## 3. Result

### 3.1. Irinotecan and OTSCC Target Determination

**Figure 2:**
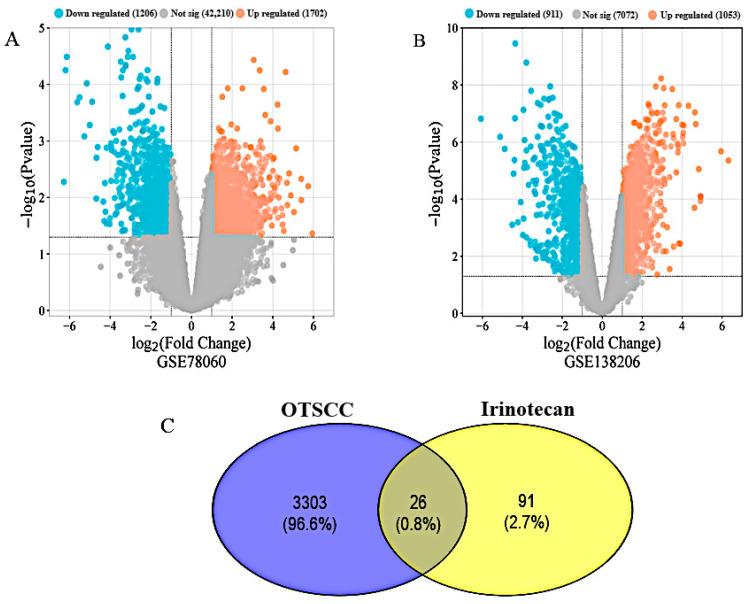
(**A**,**B**) The volcano plot shows DEGs that were elevated and downregulated in the OTSCC datasets (GSE78060 and GSE138206). The logarithmic scale (logFC) on the *X*-axis and the logarithmic scale (−log10) on the *Y*-axis represent statistical significance. (**C**) Furthermore, a Venn diagram shows all candidates who shared common targets (26 DEGs) with Irinotecan and OTSCC.

Numerous bioinformatics studies were conducted to identify the genes connected to OTSCC and the drug Irinotecan. In total, 117 genes associated with Irinotecan were found when we searched the Drug Bank, STITCH, and Swiss Target Prediction and Binding databases. Additionally, the study of the data from GSE78060 and GSE138206 yielded several DEGs. The GSE78060 and GSE138206 expression profile data were screened with the limma software using cut-off criteria and resulted in 2270 and 1412 DEGs, respectively. Furthermore, the volcano plot in Figure 2A,B indicates the total upregulated and downregulated DEGs. Then, we pooled all genes and compared them to target genes connected to Irinotecan. Finally, using the Venn diagram in Figure 2C, we discovered 26 DEGs that were shared by Irinotecan and OTSCC.

### 3.2. Analysis of DEGs for Functional and Pathway Enrichment and Selection of Hub Protein from PPI

**Figure 3:**
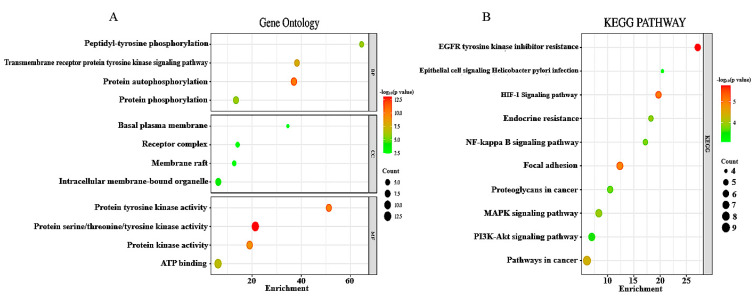
(**A**,**B**) Bubble plot diagrams demonstrating the enrichment analysis of the DEGs in Irinotecan-mediated OTSCC utilizing KEGG pathway, BP, CC, and MF. The findings of the pathway terms are ordered by *p*-value using the combined score.

**Figure 4:**
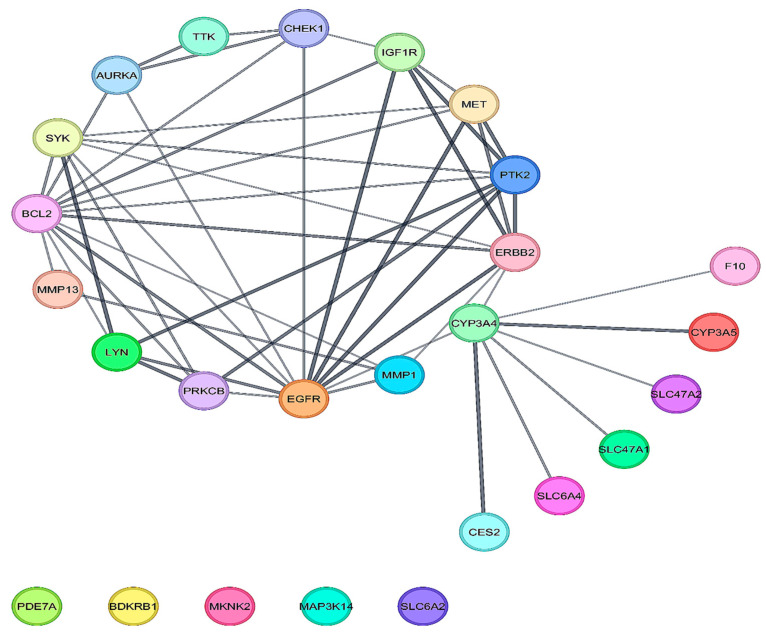
PPI network visualizations of Irinotecan’s possible treatment targets for treating OTSCC.

**Figure 5:**
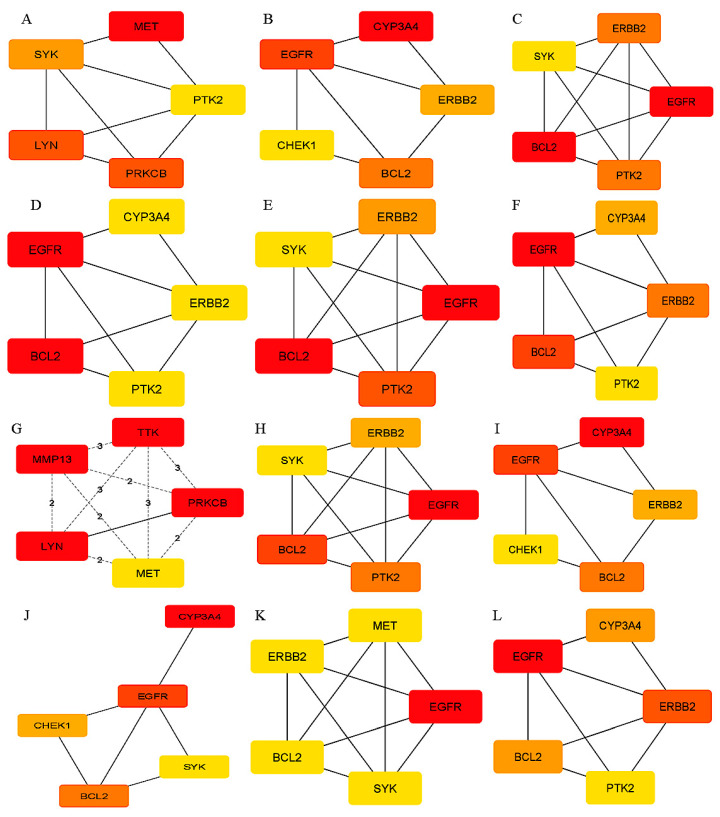
Core targets’ network diagram of Irinotecan against OTSCC by using the 12 cytoHubba algorithm: (**A**) DMNC, (**B**) Stress, (**C**) MCC, (**D**) Degree, (**E**) EPC, (**F**) Closeness, (**G**) Clustering Coefficient, (**H**) MNC, (**I**) Betweenness, (**J**) Bottleneck, (**K**) Eccentricity, and (**L**) Radiality. Hub proteins are ranked from highest to lowest using color gradients from red to yellow.

The GO of 26 DEGs was conducted using the DAVID database, and a significant threshold of *p* < 0.05 was used. According to GO analysis, the main terms enriched by the DEGs in the domain of biological function were membrane raft, protein autophosphorylation, and transmembrane receptor protein tyrosine kinase signaling pathways. The domains of cellular components, intracellular membrane-bounded organelles, and receptor complexes were the areas where DEGs were primarily concentrated. The DEGs’ molecular function was primarily connected with ATP binding and protein serine/threonine/tyrosine kinase activity, as shown in Figure 3A. The KEGG pathways enriched by the DEGs were mainly associated with EGFR tyrosine kinase inhibitor resistance, pathways in cancer, focal adhesion, and the HIF-1 signaling pathway, as presented in Figure 3B. Using the DMNC, Stress, MCC, Degree, EPC, Closeness, Clustering Coefficient, MNC, Betweenness, Bottleneck, Eccentricity, and Radiality algorithms to identify hub genes (Table 2), BCl2 and EGFR were the top two strongly linked hub genes obtained, as shown in Figure 4 and Figure 5. These two hub genes are helpful for treating OTSCC as therapeutic targets when combined with the drug Irinotecan.

### 3.3. Validation of Results via Molecular Docking

**Figure 6:**
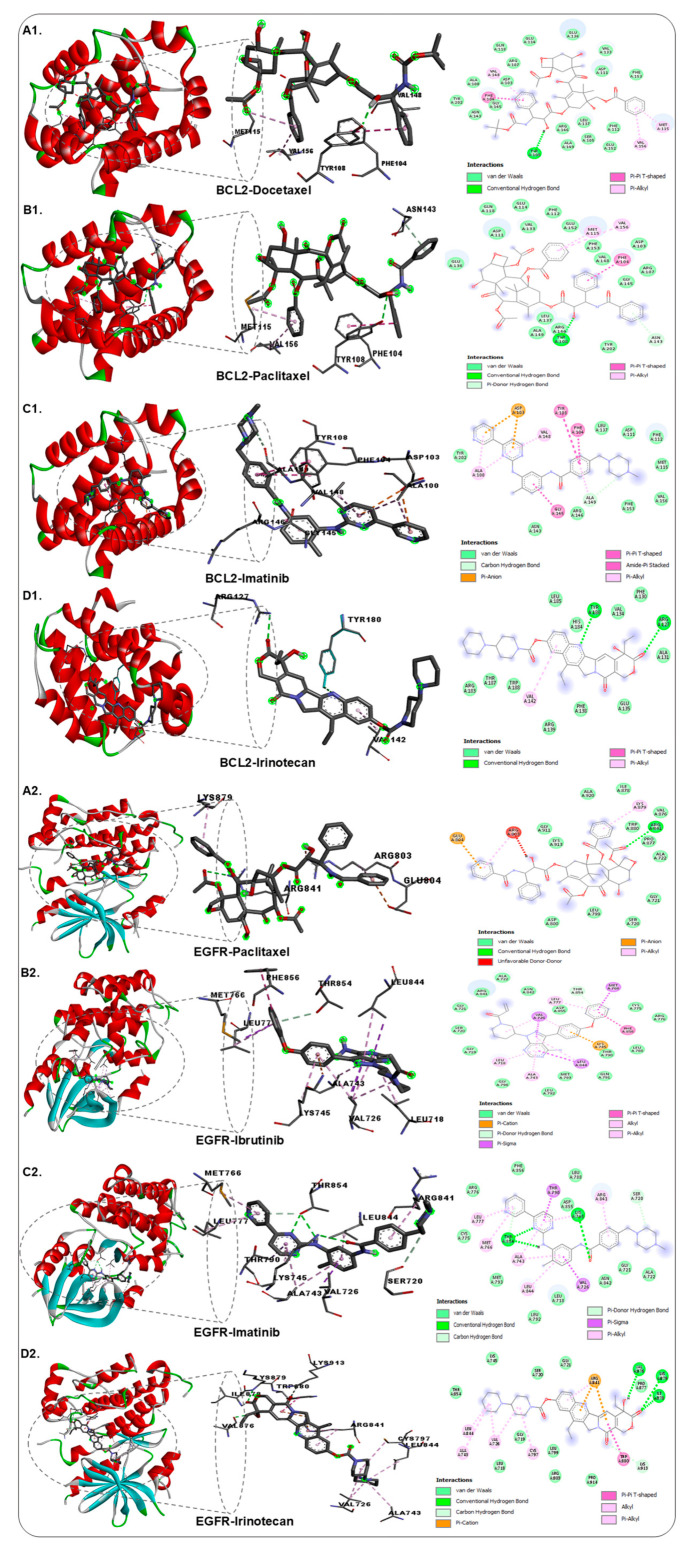
The molecular docking diagrams showing the binding capabilities of selected drugs against (**A1**–**D1**) BCL-2 and (**A2**–**D2**) EGFR proteins in both 3D and 2D views. (**A1**) Docetaxel, (**B1**) Paclitaxel, (**C1**) Imatinib, (**D1**) Irinotecan against BCL-2; (**A2**) Paclitaxel, (**B2**) Ibrutinib, (**C2**) Imatinib, and (**D2**) Irinotecan against EGFR.

The outcomes of the molecular docking demonstrated that all the binding energies between Irinotecan and the target proteins were less than 0. Utilizing molecular docking analyses, it was predicted that the drug will be able to attach to the examined protein target. The drugs Docetaxel, Paclitaxel, and Imatinib with the BCL2 protein showed binding affinity ranging between −10.5 and −9.9 kcal/mol, and the second protein EGFR, using the drugs Paclitaxel, Ibrutinib, and Imatinib, showed binding affinity ranging between −11.4 and −10.4 kcal/mol, which was higher than the control drug, Irinotecan (−9.8 and −9.6 kcal/mol, respectively) (Figure 6; Table 3).

### 3.4. MD Simulation

To validate the molecular docking results and address the dynamic behavior of ligand–receptor interactions under near-physiological conditions, 100 ns molecular dynamics (MD) simulations were performed for each protein–ligand complex involving BCL2 and EGFR receptors with Paclitaxel, Imatinib, and Irinotecan. These simulations enabled a comprehensive analysis of conformational stability, intermolecular interactions, and binding free energy estimations.

#### 3.4.1. RMSD (Root Mean Square Deviation)

**Figure 7:**
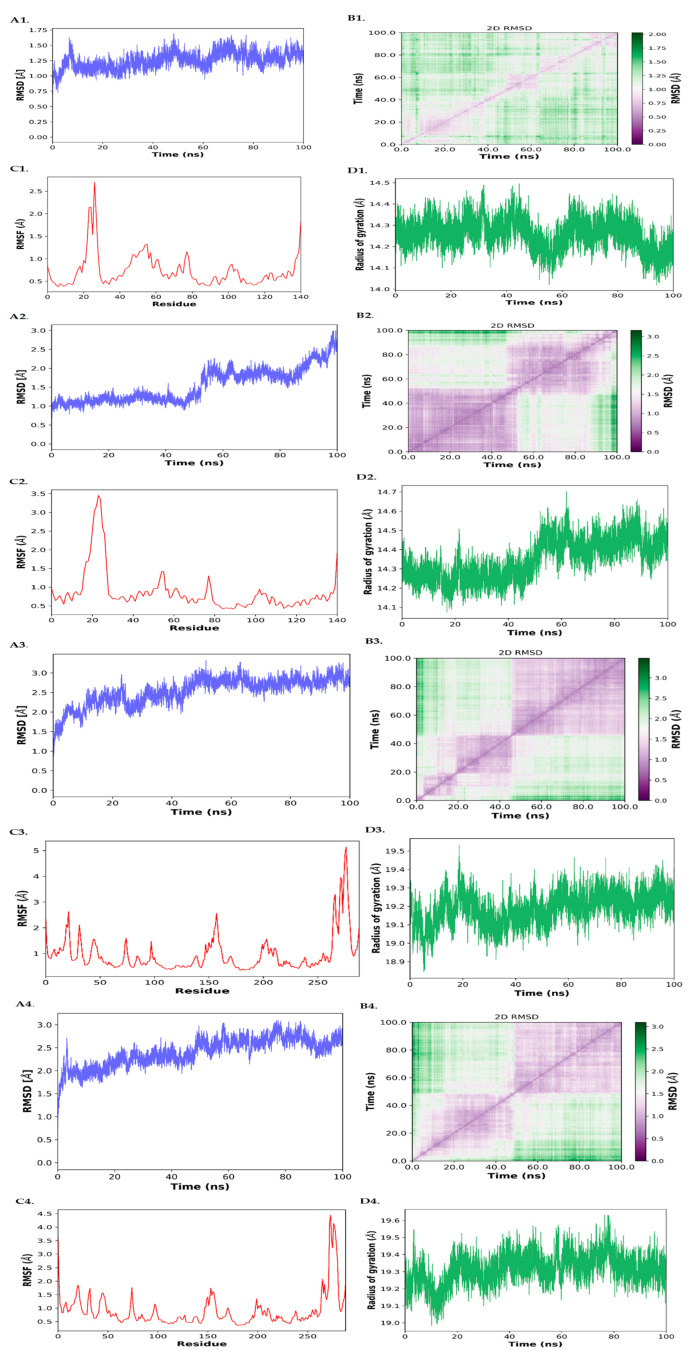
Molecular dynamics simulations of drug–protein complexes showing (**A1**–**D1**) BCL2–Imatinib, (**A2**–**D2**) BCL2–Paclitaxel, (**A3**–**D3**) EGFR–Imatinib, and (**A4**–**D4**) EGFR–Paclitaxel. For each complex, (**A**) RMSD, (**B**) 2D RMSD, (**C**) RMSF, and (**D**) radius of gyration are presented, where (**A1**–**D1**) correspond to BCL2–Imatinib, (**A2**–**D2**) correspond to BCL2–Paclitaxel, (**A3**–**D3**) correspond to EGFR–Imatinib, and (**A4**–**D4**) correspond to EGFR–Paclitaxel.

As shown in Figure 7(A1), the BCL2–Imatinib complex maintained an average RMSD of around 1.25 Å, indicating a highly stable interaction over time. In contrast, the EGFR–Imatinib complex Figure 7(A3) exhibited increased fluctuations, reaching an RMSD of ~3.0 Å toward the end of the simulation, suggesting moderate structural adjustments. The BCL2–Paclitaxel and EGFR–Paclitaxel complexes (Figure 7(A2,A4) showed moderate to higher RMSD fluctuations, with average RMSD values of ~2.5–3.0 Å, indicative of greater conformational flexibility in these systems.

#### 3.4.2. RMSF (Root Mean Square Fluctuation)

RMSF plots demonstrated that fluctuations were primarily localized at loop and terminal regions, while the binding pocket residues remained relatively stable. Notably, the BCL2–Imatinib complex (Figure 7(C1)) had lower RMSF peaks compared to Paclitaxel-bound complexes, highlighting stronger rigidity at the binding site.

#### 3.4.3. Radius of Gyration (Rg)

The compactness of each complex was analyzed using Rg (Figure 7(D1–D4)). The BCL2–Imatinib complex maintained a narrow Rg range (~14.2–14.4 Å), confirming structural compactness. Conversely, EGFR–Paclitaxel exhibited a slightly increased Rg (up to ~19.6 Å), suggesting conformational expansion during simulation.

#### 3.4.4. D RMSD Maps

These maps (Figure 7(B1–B4)) provided visual confirmation of conformational stability. BCL2–Imatinib remained clustered around low RMSD values, while EGFR–Imatinib and Paclitaxel complexes revealed wider RMSD transitions across the trajectory.

#### 3.4.5. MM/GBSA Binding Free Energy Calculations

Binding free energies were computed using MM/GBSA (Table 4) to quantitatively estimate the ligand binding strength. Among the four complexes, BCL2–Paclitaxel showed the most favorable binding energy (−39.74 ± 5.68 kcal/mol), followed by EGFR–Imatinib (−33.95 ± 6.63 kcal/mol), BCL2–Imatinib (−26.90 ± 5.41 kcal/mol), and EGFR–Paclitaxel (−20.23 ± 3.75 kcal/mol). The dominant contributors to binding energy were van der Waals and non-polar solvation terms.

## 4. Discussion

OTSCC is an aggressive oral and maxillofacial malignancy with an extremely poor prognosis [30,31]. Genetic, epigenetic, and environmental lapses work together in the occurrence of OTSCC [32]. Environmental pollutants such as aluminium chloride (AlCl_3_), persistent organic pollutants (POPs), bisphenol A (BPA), benzo[a]pyrene (BaP), and particulate matter in airborne particles may reduce the effectiveness of oral cancer chemotherapy drugs like CDDP, 5-FU, etc. [14]. Consequently, finding novel drugs to treat OTSCC is urgently needed. We selected IRT as a chemotherapeutic drug because of its high number of relapse cases in the OTSCC treatment [12,13]. Several studies have shown that after IRT treatment, more relapse cases are seen [12,13]. In the current research, we intended to uncover the vital complexities of IRT relapses in some patients in OTSCC, exploring the reasons behind relapses and potential strategies to address them through computational biology and network pharmacology methods.

Bioinformatics analysis was used to identify 3682 DEGs between OTSCC and control by retrieving gene expression data from the GEO datasets (GSE78060 and GSE138206). Further detection of a total of 117 IRN-related genes from Drug Bank, Swiss Target Prediction, Binding Databases, and STITCH was performed. After checking all DEGs, Venny 2.1.0 was used to identify the common difference gene. Finally, 26 DEGs were found between Irinotecan and OTSCC-related genes through the Venn diagram (Figure 2C). Notably, among these 26 genes, key oncogenic drivers, such as EGFR, were found to be significantly downregulated, and BCL2 was found to be significantly upregulated in OTSCC samples compared to normal tissues. Additionally, recent studies have shown that non-coding RNAs, such as miR-378, play a regulatory role in BCL2-mediated apoptosis. The overexpression of miR-378 has been reported to upregulate the Bax/BCL2 ratio and activate downstream apoptotic signals (cleaved caspase-9, caspase-3, and PARP), thereby promoting apoptosis in OSCC cells. In contrast, inhibition of miR-378 suppresses apoptosis, suggesting its involvement in the dysregulation of cell proliferation and survival in OSCC [33].

Although EGFR was downregulated in our datasets, previous evidence demonstrates that low EGFR expression frequently occurs in high-grade OSCC with more invasive behavior and EMT features [34]. However, EGFR remains a relevant therapeutic target—even in contexts of reduced expression—due to its pivotal role in downstream signaling pathways and potential residual activity.

To investigate the link between DEGs and the KEGG pathway, Gene Ontology analysis was performed. The GO analysis provided insight into the fact that the DEGs played a role in pathways such as protein phosphorylation, protein autophosphorylation, protein serine/threonine/tyrosine kinase activity, etc. Numerous physiological functions, such as signaling, cell division, consumption of energy, and cellular motility, are disturbed by protein phosphorylation, and these activities all contribute to the development of tumours [35]. Additionally, protein phosphorylation on tyrosine residues is important in the regulation of critical pathways that contribute to the development of OTSCC [36]. IRN almost completely suppressed the protein’s phosphorylation, indicating the drug might interfere with a variety of signaling pathways. Cancer develops because of environmental factors that inhibit apoptosis and resistance to chemotherapy and radiation therapy, both of which destroy cancer cells [37,38,39]. Additionally, the KEGG pathways for DEGs included cancer-related pathways like HIF-1 signaling and EGFR tyrosine kinase inhibitor resistance. Furthermore, the PPI network reveals linked hub genes using twelve distinct algorithms (Table 2). The presence of BCL2 and EGFR in these pathways and networks suggests that they are important in the development of OTSCC. B cell lymphoma 2 family (BCL2) gene activation, which promotes carcinogenesis through BCL2-mediated apoptosis, is a frequent sign of cancer [40]. The BCL-2 transcription is induced by proliferative cytokines or signaling pathways like the Ras-mediated pathway and PI3K/AKT [41,42]. OTSCC cells are efficiently targeted by Bcl-2 inhibition because it reduces proliferation and induces cell death [43]. BCL-xL and BCL-2 (anti-apoptotic proteins) were shown to be more abundant in cancer cell lines treated with chemotherapeutic drugs and bisphenol A [44,45]. So, there is a need to explore a variety of drugs for targeting BCL-2. Epidermal Growth Factor Receptor (EGFR) is a tyrosine kinase (TK) receptor that is connected to radiation resistance and prognosis in HNSCC [46,47]. Since EGFR is raised in over 90% of HNC, it offers potential as a therapeutic target [48,49]. Since all OTSCCs were shown to have elevated EGFR levels, this cancer type is particularly attractive for research into potential novel treatments that target the EGFR receptor [50].

The concise list of FDA-approved drugs was extracted from the DGIdb (http://www.dgidb.org) database, which is related to the BCL2 and EGFR proteins. After molecular docking, Docetaxel, Paclitaxel, and Imatinib showed binding affinity ranging between −10.5 and −9.9 kcal/mol against the BCL2 protein. The drugs Paclitaxel, Ibrutinib, Imatinib, Ponatinib, Ibrutinib, Sorafenib, and Etoposide showed a binding affinity ranging between −11.4 and −9.7 kcal/mol against EGFR protein, which was higher than the control drug Irinotecan, −9.8 and −9.6 kcal/mol, against BCL2 and EGFR (Table 3). Paclitaxel and Imatinib were commonly used in targeting with high binding affinity to both the proteins, i.e., BCL2 and EGFR. According to the literature, Paclitaxel and Imatinib interfere with the EGFR and apoptosis-related pathways [51,52]. Previous studies have shown that using Paclitaxel and Imatinib along with other cancer treatments has considerable effects [53,54,55,56].

The initial molecular docking data suggested strong binding affinities of Imatinib and Paclitaxel with BCL2 and EGFR proteins, yet these findings alone could not fully capture the dynamic nature of protein–ligand interactions. To address this, 100 ns MD simulations were performed for each complex to evaluate structural stability, conformational flexibility, and binding energy dynamics. The RMSD and Rg analyses for BCL2–Imatinib (Figure 7(A1,D1) revealed remarkable stability and compactness, validating the docking pose under physiological conditions. Similarly, RMSF data (Figure 7(C1)) indicated minimal fluctuations at the binding site, supporting stable ligand accommodation. In contrast, the EGFR–Imatinib and EGFR–Paclitaxel complexes exhibited higher RMSD and Rg deviations (Figure 7), implying greater structural reorganization, which may influence binding efficacy. Importantly, the MM/GBSA results corroborated these dynamic findings, showing that BCL2–Paclitaxel had the most favorable estimated binding energy (Table 4). Despite lower electrostatic contributions, the overall binding energy was stabilized by strong van der Waals and non-polar solvation forces. This suggests that Paclitaxel may act as a viable repurposed therapeutic candidate targeting BCL2 in OTSCC. Moreover, EGFR–Imatinib also showed promising interaction profiles, supported by moderate RMSD and favorable binding free energy, indicating Imatinib as a potential EGFR-targeting agent. These results clearly demonstrate that molecular docking alone cannot sufficiently predict ligand stability or binding efficacy. Our integration of 100 ns MD simulations and MM/GBSA analysis strengthens the reliability of the in silico predictions and enhances the translational significance of Paclitaxel and Imatinib as potential repurposed agents in OTSCC.

In conclusion, our study identifies Irinotecan-mediated biomarkers in OTSCC and presents a scientific bioinformatics analysis of DEGs that may be linked to the development of OTSCC. These findings are crucial for creating accurate predictions and innovative therapy options for OTSCC, especially in chemoresistance caused by environmental pollutants. Then, we performed logarithmic correction to all datasets to reduce the impact of outliers. Furthermore, this study may provide insights into the treatment of novel diagnostic and pharmacological targets, as well as inform the development of disease, all of which might be incredibly beneficial for long-term healthcare.

## 5. Study Limitations

This study provides an integrative in silico framework combining gene expression analysis, molecular docking, and molecular dynamics (MD) simulations to explore the chemoresistance landscape in OTSCC and predict Paclitaxel and Imatinib as potential inhibitors of BCL2 and EGFR. However, there are several limitations to acknowledge. First, the gene expression datasets used were limited in sample size and derived from publicly available repositories, which may not fully represent patient heterogeneity or disease complexity. Second, while molecular docking offers insights into ligand–receptor binding affinities, it provides only a static view and does not account for the dynamic nature of protein-ligand interactions under physiological conditions. To address this, MD simulations were employed. Our findings are promising: further laboratory-based experiments are necessary to validate the therapeutic relevance of Paclitaxel and Imatinib in overcoming chemoresistance in OTSCC.

## 6. Conclusions

Environmental pollutants could collaborate in chemoresistance by affecting apoptosis function, activating various pathways such as MAPK/ERK, NF-кB, and PI3K/AKT; abolition of p53 signaling; drug efflux; and antioxidant enzyme activity. All these mechanisms interact and are resistant to chemotherapeutic drugs, as well as demonstrate chemoresistance, which increases with specific cancer types. Therefore, to achieve effectiveness, we must administer therapies at higher doses or combine different drugs. In the current work, we investigated the signaling pathways linked to DEGs and a possible key candidate gene in OTSCC through bioinformatics and system biology approaches. The regulation of protein phosphorylation, the PI3K-Akt signaling pathway, protein tyrosine kinase activity, and cancer pathways critical for OTSCC progression have all been found to be concentrated in hub genes. Two of the hub genes, EGFR and BCL2, showed strong network connectivity in cytoHubba’s twelve-algorithm screening. After molecular docking, Docetaxel, Paclitaxel, and Imatinib showed a binding affinity ranging between −10.5 and −9.9 kcal/mol against the BCL2 protein. The drugs Paclitaxel, Ibrutinib, Imatinib, Ponatinib, Ibrutinib, Sorafenib, and Etoposide showed a binding affinity ranging between −11.4 and −9.7 kcal/mol against the EGFR protein, which was higher than the control drug, Irinotecan (−9.8 and −9.6 kcal/mol), against BCL2 and EGFR. Paclitaxel and Imatinib were commonly used in targeting, exhibiting high binding affinity to both the proteins, i.e., BCL2 and EGFR. To validate binding stability, molecular dynamics simulations were performed, revealing that BCL2–Imatinib maintained stable conformations, while BCL2–Paclitaxel showed stronger binding free energy via MM/GBSA. Similar trends in EGFR complexes support Paclitaxel and Imatinib as promising repurposed therapies for OTSCC. This research provides a better understanding of the pathophysiology of OTSCC and its underlying molecular foundations, which opens up possibilities for uncovering novel therapeutic targets to treat OTSCC by leveraging selected prospective genes and pathways. This work paves the way for sustainable healthcare and tackling environment-related chemoresistance; thus, it is instrumental in treating OTSCC.

## Figures and Tables

**Table 1:** Summary of GEO datasets (GSE78060 and GSE138206) used for differential expression analysis in OTSCC, including sample types, size, inclusion/exclusion criteria, and microarray platform.

Dataset	Sample Type	Number of Samples	Inclusion Criteria	Exclusion Criteria	Platform
GSE78060	OTSCC Tissue	26 Tumor, 4 Normal	Histologically confirmed OTSCC, untreated patients, transcriptomic data of GPL570	Incomplete data; less than 10 samples	GPL 570, [HG-U133_Plus_2] Affymetrix Human Genome U133 Plus 2.0 Array
GSE138206	OTSCC Tissue	5 Tumor, 5 Normal	Histologically confirmed OTSCC, untreated patients, transcriptomic data of GPL570	Incomplete data; less than 10 samples	GPL 570, [HG-U133_Plus_2] Affymetrix Human Genome U133 Plus 2.0 Array

**Table 2:** List of genes that are found in different topologies of cytoHubba.

Topology	Occurrence	Genes
BottleNeck, Betweenness, Degree, Closeness, EcCentricity, EPC, Radiality, MCC, MNC, Stress	2	BCL2, EGFR
Closeness, Betweenness, EcCentricity, Degree, MCC, EPC, MNC, Radiality, Stress	1	ERBB2
DMNC, Closeness, EPC, Degree, MCC, Radiality, MNC	1	PTK2
EPC, BottleNeck, DMNC, EcCentricity, MNC, MCC	1	SYK
BottleNeck, Betweenness, Degree, Closeness, Stress, Radiality	1	CYP3A4
Clustering Coefficient, DMNC, EcCentricity	1	MET
Betweenness, BottleNeck, Stress	1	CHEK1
Clustering Coefficient, DMNC	2	PRKCB, LYN
Clustering Coefficient	2	MMP13, TTK

**Table 3:** List of drugs with binding affinity.

Drugs	BCL2	EGFR
Docetaxel	−10.5	−8.6
Paclitaxel	−10.3	−11.4
Imatinib	−9.9	−10.4
Irinotecan	−9.8	−9.6
Ponatinib	−9.6	−10.2
Ibrutinib	−9.1	−11
Teniposide	−9	−8.4
Sorafenib	−8.7	−9.9
Epirubicin	−8.7	−8.9
Temsirolimus	−8.6	−9.3
Pemetrexed	−8.5	−9.3
Doxorubicin	−8.3	−8.9
Dasatinib	−8.1	−8.8
Everolimus	−8.1	−7.9
Raltitrexed	−8.1	−7.6
Etoposide	−7.9	−9.7
Methylprednisolone	−7.7	−7.9
Sirolimus	−7.5	−7.1
Sunitinib	−7.3	−8.8
Bortezomib	−7.3	−8.3
Bosutinib	−7.3	−7.5
Vincristine	−7.3	−6.9
Tretinoin	−7	−8.7
Mitoxantrone	−6.5	−8.5
Floxuridine	−6.2	−6.3
Streptozocin	−6.1	−6.1
Gemcitabine	−6	−6.5
Temozolomide	−6	−5.7
Decitabine	−5.6	−6.1
CARBOPLATIN	−5.5	−5.1
Fluorouracil	−4.9	−5.3

**Table 4:** Binding free energies calculated using the MM/GBSA method.

Sr. No.	Protein–Ligand	Van der Waals Energy (kcal/mol)	Electrostatic Energy (kcal/mol)	Polar Solvation Energy (kcal/mol)	Non-polar Solvation Energy (kcal/mol)	Estimated Binding Energy (kcal/mol)
1	BCL2–Imatinib	−44.15 ± 5.73	−119.29 ± 14.24	142.17 ± 14.74	−5.62 ± 0.63	−26.90 ± 5.41
2	BCL2-Paclitaxel	−59.96 ± 5.90	−14.33 ± 6.45	42.42 ± 6.48	−7.86 ± 0.75	−39.74 ± 5.68
3	EGFR-Imatinib	−53.52 ± 3.83	−20.97 ± 9.63	47.51 ± 8.03	−6.97 ± 0.26	−33.95 ± 6.63
4	EGFR-Paclitaxel1	−36.05 ± 2.52	−21.55 ± 15.62	42.07 ± 13.66	−4.71 ± 0.64	−20.23 ± 3.75

## Data Availability

The datasets used and/or analyzed during this study are available from the corresponding author upon reasonable request.
